# Estimation of Genetic Parameters by Single-Trait and Multi-Trait Models for Carcass Traits in Hanwoo Cattle

**DOI:** 10.3390/ani9121061

**Published:** 2019-12-02

**Authors:** Swati Srivastava, Bryan Irvine Lopez, Sara de las Heras-Saldana, Jong-Eun Park, Dong-Hyun Shin, Han-Ha Chai, Woncheol Park, Seung-Hwan Lee, Dajeong Lim

**Affiliations:** 1 National Institute of Animal Science, Rural Development Administration, Wanju 55365, Korea; swati051@gmail.com (S.S.); irvinelopez@korea.kr (B.I.L.); jepark0105@korea.kr (J.-E.P.); wcpark1982@korea.kr (W.P.); hanha@korea.kr (H.-H.C.); 2 School of Environmental and Rural Science, University of New England, Armidale 2351, NSW, Australia; sdelash2@une.edu.au; 3 Department of Animal Biotechnology, Chonbuk National University, Jeonju 54896, Korea; sdh1214@gmail.com; 4 Department of Animal Science and Biotechnology, Chungnam National University, Daejeon 34134, Korea

**Keywords:** genetic parameters, carcass traits, Hanwoo cattle, single-trait, multi-trait

## Abstract

**Simple Summary:**

Genetic parameters play an important role in designing a breeding program. Many software and methods are used to estimate genetic parameters in livestock population. Multi-trait models are efficiently used these days for productive traits, reproductive traits, milk traits, etc. These models are more useful in the case of low heritability trait and with missing phenotypes. In this study, Hanwoo cattle, an indigenous breed from South Korea, is studied for four carcass traits (back fat thickness, carcass weight, eye muscle area, and marbling score). Single-trait and multi-trait models are constructed using BLUPF90 software to estimate variance components. In addition, the effect of genetic correlations among traits is scrutinized in multi-trait models for these traits.

**Abstract:**

Hanwoo breed is preferred in South Korea because of the high standards in marbling and the palatability of its meat. Numerous studies have been conducted and are ongoing to increase the meat production and quality in this beef population. The aim of this study was to estimate and compare genetic parameters for carcass traits using BLUPF90 software. Four models were constructed, single trait pedigree model (STPM), single-trait genomic model (STGM), multi-trait pedigree model (MTPM), and multi-trait genomic model (MTGM), using the pedigree, phenotype, and genomic information of 7991 Hanwoo cattle. Four carcass traits were evaluated: Back fat thickness (BFT), carcass weight (CWT), eye muscle area (EMA), and marbling score (MS). Heritability estimates of 0.40 and 0.41 for BFT, 0.33 and 0.34 for CWT, 0.36 and 0.37 for EMA, and 0.35 and 0.38 for MS were obtained for the single-trait pedigree model and the multi-trait pedigree model, respectively, in Hanwoo. Further, the genomic model showed more improved results compared to the pedigree model, with heritability of 0.39 (CWT), 0.39 (EMA), and 0.46 (MS), except for 0.39 (BFT), which may be due to random events. Utilization of genomic information in the form of single nucleotide polymorphisms (SNPs) has allowed more capturing of the variance from the traits improving the variance components.

## 1. Introduction

Korean beef cattle Hanwoo is one of the four aboriginal breeds among Hanwoo, Chikso, Heugu and Jeju Black. Its farming is streamlined due to high customer demand and competition with other types of meat. Therefore, emphasis is laid on the improvement of carcass traits such as back fat thickness (BFT), carcass weight (CWT), eye muscle area (EMA), and marbling score (MS) to enhance the selection of animals for breeding programs. [1]. Utilization of DNA markers can enhance genetic gain. These DNA markers can be used in predicting breeding values, and hence, can lead to accurate selection of animals [2]. Genomic prediction via single nucleotide polymorphisms (SNPs) and phenotypes is an emerging field which includes animal and plant breeding, risk prediction in human medicine, and forensics. To help in conducting these breeding programs, estimation of genetic parameters plays an important role, and they are generally evaluated based on single-trait models. However, these do not account for the covariance among traits and may result in inaccurate estimates of breeding values, leading to selection bias. Multi-trait models include multiple genetic effects within the same traits or multiple traits within same groups. These are more explicit compared to single-trait analysis, as they consider the information provided by genetic correlation while predicting the breeding value and heritability of any trait [3,4,5,6,7]. These correlations specify the relationship among traits. Multiple-trait genomic selection can increase accuracy of prediction [4]. In addition, using multi-trait models can provide reliable and unbiased estimates of genetic parameters [2]. Various studies were conducted focusing on single-trait analyses utilizing the Bayesian method, GBLUP, ssBLUP, etc., to evaluate the best model in the estimation of genetic parameters.

Many traits are genetically correlated, such as reproductive traits, milk yielding traits, etc., and have different heritabilities when calculated in single-trait and multi-trait models. Multi-trait models can improve the estimations of traits with low-heritability trait or small population size [5,8]. Many studies were conducted comparing single-trait and multi-trait analysis on productive, reproductive, and milk traits using different software and methods. A study on 1567 Holstein was conducted for reproductive and productive traits through DMU software [9]. Another work was conducted on bulls through GBLUP, considering only genomic prediction utilizing QTL information [5]. To our knowledge, multi-trait models are not used to improve the estimation of genetic parameters for carcass traits in Hanwoo. Moreover, previous studies faced the limitation of small sample size that has available genomic information. Better estimation of variance components that consider the genetic correlations of important traits for breeding objectives will help in setting a total merit index for accurate evaluation. Therefore, the main objective of this study was to compare genetic parameters estimated by four different models, i.e., single-trait pedigree model (STPM), single-trait genomic model (STGM), multi-trait pedigree model (MTPM), and multi-trait genomic model (MTGM), for four carcass traits (BFT, CWT, EMA, and MS) in a large population of Hanwoo, Korean beef cattle.

## 2. Materials and Methods

### 2.1. Korean Hanwoo Cattle Data

Phenotypic data for carcass traits, i.e., back fat thickness (BFT), carcass weight (CWT), eye muscle area (EMA), and marbling score (MS), were collected from 7991 commercial Hanwoo cattle, which were slaughtered at the age of approximately 30 months (Table 1). All procedures followed in this study were according to animal health and welfare guidelines approved by the Animal Care and Use Committee of the National Institute of Animal Science (NIAS), Rural Development Administration (RDA), South Korea (2018-293). Apart from animal, parent sire, and parent dam information, other information such as birth year, birth month, slaughter year, slaughter place, age, sex, and herd was also provided. Pedigree data were available for 38,731 animals. Animals with missing sire and dam information were marked as 0 to avoid null value. Phenotypic data comprised back fat thickness (BFT) in millimeters (mm), carcass weight in kilograms (kg), marbling score (MS) with grading 1–9, and eye muscle area (EMA) in centimeter square (cm^2^). There were no missing phenotypes.

Single nucleotide polymorphism (SNP) data comprised 53,866 SNPs from 7991 animals. Tissue samples were genotyped using Illumina Bovine SNP50k. Quality check for the SNP data was done by the PREGSF90 program of BLUPF90. In total, 143 SNPs were removed which had less than 0.90 call rate, 488 SNPs with allele frequency <0.01 were removed, and another 324 monomorphic SNPs were also eliminated. All animals had more than 0.90 call rate and there were no animals removed after using a Hardy–Weinberg equilibrium threshold of 0.15 (default value in BLUPF90). Finally, 48,984 SNPs and 7991 animals were left for further analysis.

### 2.2. Statistical Analysis

As our data comprised much information therefore, statistical significance of fixed effects needed to be analyzed. This was calculated through the ANOVA package present in R program (version 3.4.4, R Core Team, 2013). Significant effects were used as fixed effects in further analysis. HYM (herd birth year month), slaughter year month, slaughter place, and sex were taken as categorical values, whereas age was taken as a continuous value [10,11]. Through the analysis, it was observed that age, sex, and HYM had a significant effect on the studied traits. Hence, all the models were built taking age, sex, and HYM (herd birth year and month) as fixed effects. All calculations and manipulation of the data were performed using the R program (version 3.4.4, R Core Team, 2013).

### 2.3. Models

BLUPF90, a software that comprises a family of program in Fortran 90/95 for mixed model computations in animal breeding [12]. In this analysis, missing values were allowed and replaced with 0. The cleaning and processing of the data was done automatically by PREGSF90. First, the data were renumbered and variance was estimated using RENUMF90. Then, the AIREMLF90 (average information restricted maximum likelihood) [13,14] was used to estimate variance components and calculate heritability and genetic correlations. These various components were then used to predict estimated breeding values (EBV). Single-trait (ST) and multi-trait (MT) models (comprising all four traits together) were built for both pedigree (STPM: Single-trait pedigree model) and genomic (STGM: Single-trait genomic model) data (Table 2). These models were built to compare variance components and estimated breeding value (EBV) estimated by various models [15]. These models included fixed effects of contemporary group (HYM), sex, and age.

(i) Single-Trait Model.

In the single-trait model (ST), genetic parameters were estimated for each single-trait (BFT, CWT, EMA, and MS). A comparative study was also done between pedigree and genomic models. Hence, two single-trait animal models were built—one with pedigree and phenotype data (STPM) and the other with pedigree, phenotype, and genomic information (STGM). A linear mixed model of BLUPF90 software was used for single-trait analysis as in Equation (1):

y = X*β* + Z*α* + r (1)

where y is the vector of phenotypic observations or traits (BFT, CWT, EMA and MS); *β* is the vector of all fixed effects calculated above; α is the vector of random additive genetic effect for each animal in STPM and STGM; r is the vector of random residual; and X and Z are the incidence matrices for each corresponding effect. Variance components comprising additive genetic variance and residual variance can be found in log file generated by software. EBV (estimated breeding value for pedigree model) and GEBV (genomic estimated breeding value for genomic), along with s.e (standard error), were also predicted.

(ii) Multi-Trait Model

A multi-trait model (MT) was built considering all the four carcass traits (BFT, CWT, EMA, and MS) through BLUPF90 software. A comparative study was done between multi-trait pedigree (MTPM) and multi-trait genomic (MTGM) models. Hence, two multi-trait animal models were built—one with pedigree and phenotype data and the other with pedigree, phenotype, and genomic information. The models and inferences were similar to the pedigree-based models described above, with the exception that this model uses a genomic relationship matrix (G) created from the SNP markers instead of the pedigree-based relationship (A). Following VanRaden [14], the G matrix was constructed using all markers as in Equation (2):

(2) $$G=\frac{{MM}^{\prime }}{\sum 2p_{i}\left(1-p_{i}\right)}$$

where M is a matrix of centered genotypes, and $p_{i}$ is the second allele frequency at locus i. The Variance component comprising additive genetic variance and residual variance was predicted in log file. EBV and GEBV, along with s.e, were obtained in solution file.

### 2.4. Variance Component and Heritability Estimation

EBVs (estimated breeding values) were predicted from phenotypic data by both STPM and MTPM. The GEBVs were predicted by the GM using genotype (SNPs) and phenotype data for STGM and MTGM. The variance component comprising genetic variance and residual variance was estimated.

Heritability was calculated as in Equation (3):

(3) $$h^{2\;}=\frac{\sigma_{g}^{2}}{\sigma_{g}^{2}+\;\sigma_{r}^{2}}\;$$

where $h^{2\;}$ is heritability of a trait, $\sigma_{g}^{2}$ is genetic variance, and $\sigma_{r}^{2}$ is residual variance obtained. A comparison was made on the heritability predicted by all models.

### 2.5. Correlation

In actual breeding, many traits that are correlated. Considering correlation among the traits might result in more accurate GEBV, the genetic correlations were calculated based on covariance as in Equation (4):

(4) $$r_{g}=\;\frac{{\mathrm{cov}}_{g}}{\sqrt{V_{g1}V_{g2}}}$$

where r_g_ is the genetic correlation, cov_g_ is genetic covariance between trait 1 and trait 2, V_g1_ is the genetic variance of trait 1, and V_g2_ is genetic variance of trait 2.

## 3. Results

### 3.1. Trait Summary and Heritability

**Figure 1:**
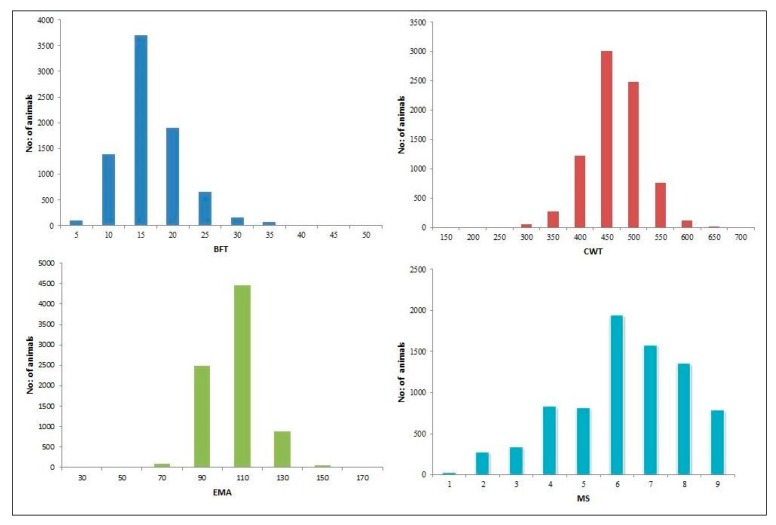
Frequency distribution for the carcass traits: BFT (back fat thickness), CWT (carcass weight), EMA (eye muscle area), and MS (marbling Score) for 7991 Hanwoo cattle.

The descriptive statistics for the carcass traits analyzed in this work are listed in Table 1, along with the frequency distribution of the phenotypic data in Figure 1. In the analyzed population the BFT ranged from a minimum of 2 to 47 mm, the values for CWT from 159 to 692 kg, MS grades from 1 to 9, and EMA from 34 to 156 cm^2^ for 7991 animals.

#### 3.1.1. Single-Trait Model

Variance components, i.e., additive genetic variance and residual variance, were estimated for both STPM and STGM. Heritability was calculated from the variance components obtained through STPM. It was 0.40, 0.33, 0.36, and 0.35 for BFT, CWT, EMA, and MS, respectively (Table 3). There was a notable change in the estimated genomic heritability calculated through STGM when combining pedigree, phenotypes, and genotypes of the animals. The calculated heritabilities were 0.39, 0.39, 0.39, and 0.46 for BFT, CWT, EMA, and MS, respectively.

#### 3.1.2. Multi-Trait Model

The multi-trait (MT) models were constructed considering all four traits together for both PM and for GM. A comparative study was conducted between MTPM and MTGM (Table 3). A slight increase in heritability was observed for the multi-trait pedigree model (MTPM) as compared to heritability obtained for the single-trait pedigree model (STPM). The results show heritabilities of 0.41, 0.34, 0.37, and 0.38 for BFT, CWT, EMA, and MS, respectively, for MTPM. Contrary, there was no significant increase in heritability for MTGM as compared to STGM. Instead, a slight increase in genetic variance was observed for BFT and EMA, with similar genetic variance for MS and a decreased variance for CWT. Residual variance was slightly decreased for BFT and EMA, similar residual variance for MS, and an increased variance for CWT, accounting for similar heritability for both STGM and MTGM.

### 3.2. Genetic and Phenotypic Correlation

**Figure 2:**
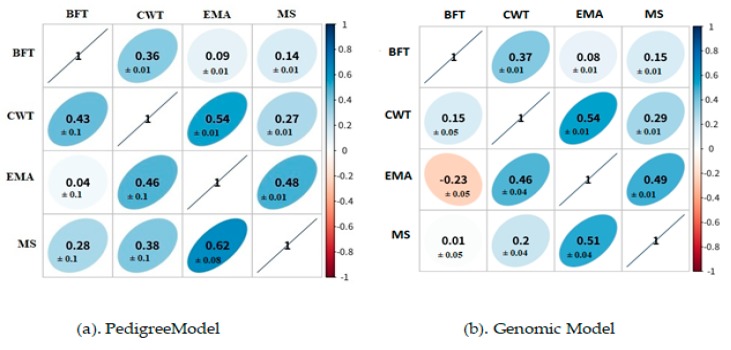
Genetic correlation and phenotypic correlation among traits for (**a**) the pedigree model and (**b**) the genomic model. Above diagonal is the phenotypic correlation and below diagonal is the genetic correlation for four carcass traits: BFT (Back fat thickness), CWT (carcass weight), EMA (Eye Muscle area), MS (Marbling score).

The genetic correlations were calculated by the multi-trait animal model using BLUPF90 software. As correlations always occur between two or more traits, the multi-trait model was used to compute genetic correlation among the four traits. Results obtained for both genetic and phenotypic correlation are shown in Figure 2. For the pedigree and genetic models, a significant phenotypic correlation of 0.54 ± 0.01was found between CWT and EMA. Whereas a genetic correlation of 0.62 ± 0.08 was found between EMA and MS for the pedigree model, and 0.51 ± 0.04 was found between EMA and MS for the genomic model. A negative correlation of −0.23 ± 0.05 was also observed between BFT and EMA for the genomic model.

## 4. Discussion

Genetic parameters play a significant role in designing a breeding program. Therefore, a study was conducted to evaluate single-trait and multi-trait models and to compare pedigree and genomic models to highlight the significance of using SNPs. Variance components were estimated through the above constructed model. Until now, many methods and software were used to estimate breeding value. One such software is WOMBAT [16], in which 10,286 Hanwoo cattle were used with the pedigree of 35,268 animals with no genomic information. They reported heritability for three traits: BFT (0.45), CWT (0.29), and MS (0.62). It has been observed that heritability for CWT is reported more in our work, whereas estimated heritability for BFT and MS was less than those estimated in the mentioned study. Another study was conducted by the GWAS (genome-wide association study) method on 1011 Hanwoo cattle and reported heritability of BFT (0.40), CWT (0.33), EMA (0.41), and MS (0.50) [17], which lies close to our estimated genomic parameters. Park et al. reported heritabilities of BFT (0.50), CWT (0.30), EMA (0.42), and MS (0.63), where basic statistics were analyzed through SAS 9.02 and genetic parameters were estimated with ASReml [18]. Another work reported the heritabilities of BFT (0.29), CWT (0.51), EMA (0.45), and MS(0.22) using the Restricted maximum likelihood (REML) procedure on approximately 1100 Hanwoo cattle slaughtered at –30 months stage [19]. As compared to this work, there was an increase in heritability for BFT and MS. Heritability of carcass traits such as EMA, BFT, and MS in Japanese black cattle (Wagyu) was estimated at 0.02, 0.15, and 0.49, respectively [20]. A study conducted on another breed, i.e., 1756 Nellore cattle, estimated heritability for BFT (0.08), CWT (0.17), and EMA (0.20) using Bayesian inference in BLUPF90 family programs [21].

In this work, BLUPF90 software was used to construct various models using pedigree, phenotype, and genomic information. Four different models were created and compared for four traits. An increased heritability was estimated by the genomic model (GM) as compared to the pedigree model (PM), i.e., BFT (0.39), CWT (0.39), EMA (0.39), and MS (0.46), for both single-trait and multi-trait models.

Genetic correlation is the proportion of variance that two traits share due to genetic causes. It can be positive or negative; a positive genetic correlation helps in improvement of the two traits, whereas a negative correlation implies that selection of one trait can cause deterioration of both traits [7]. It also helps in understanding the linkage between the genetically correlated traits. An increase in heritability for the multi-trait model as compared to the single-trait model may be due to the incorporation of information occurring by correlation among the traits in the multi-trait model. It can be observed that genetic correlation among traits reached higher values than published in literature [18,22] with approximately similar phenotypic correlation between BFT–CWT, CWT–EMA, and BFT–MS. Further, the high genetic correlation between traits is advantageous in the multi-trait model for low heritability traits, as it helps in understanding how closely or distantly traits are associated with each other [23,24]. A strong correlation was observed between EMA–MS (pedigree = 0.63, genomic = 0.51). Interestingly, an increase in heritability estimates indicates that the multi-trait pedigree model can be a better than the single-trait pedigree model due to correction of selection bias by the multi-trait model, as it uses correlated trait information [9].

There was no increase in heritability for any traits from STGM to MTGM. This may be because the heritabilities are not low for the traits, and all traits have full phenotype information available. An increased genetic parameter in the genomic model infers that genomic information, i.e., SNPs, plays a vital role in the prediction of breeding values and estimation of heritability in a population. It is because these SNPs can comprehend Mendelian sampling, i.e., heritability in populations, based on the expected proportion of genes shared between different types of relatives [6,18], and this accounts for similar heritability for STGM and MTGM.

## 5. Conclusions

Accurate estimation of genetic parameters plays an essential strategic step for breeding programs, which can help in selective breed improvement in a population. Increased heritabilities obtained by MTPM as compared to STPM highlights the significance of correlation. Whereas, increased heritability of the genomic model over the pedigree model infers a significant role of SNPs in breeding. This can be seen through noticeable increased values of heritability and breeding values for marbling score. Genetic parameters estimated through this work can be used for developing future breeding programs involving genomic prediction methods.

## Figures and Tables

**Table 1:** Descriptive statistics for BFT (back fat thickness), CWT (carcass weight), EMA (eye muscle area), and MS (marbling score), respectively, in Hanwoo cattle for 7991 cattle.

Carcass Traits	Mean	SD	Min	Max	Number of Animals
BFT (mm)	14.48	4.98	2	47	7991
CWT (kg)	440.94	52.36	159	692	7991
EMA (cm^2^)	96.29	12.13	34	156	7991
MS (1–9)	6.23	1.81	1	9	7991

**Table 2:** Information used by four different statistical models.

Model	Pedigree	Phenotype	Genotype	Single Trait	All Trait
STPM	Yes	Yes	No	Yes	No
MTPM	Yes	Yes	No	No	Yes
STGM	Yes	Yes	Yes	Yes	No
MTGM	Yes	Yes	Yes	No	Yes

**Table 3:** Heritability and variance component calculated for single-trait and multi-trait model on pedigree and genomic data using AIREML in BLUPF90.

Heading	Pedigree Model (PM)								Genomic Model (GM)							
	BFT		CWT		EMA		MS		BFT		CWT		EMA		MS	
	ST	MT	ST	MT	ST	MT	ST	MT	ST	MT	ST	MT	ST	MT	ST	MT
$h^{2}$	0.40	0.41	0.33	0.34	0.36	0.37	0.35	0.38	0.39	0.39	0.39	0.39	0.39	0.39	0.46	0.46
s.e	0.06	0.06	0.05	0.04	0.05	0.06	0.05	0.05	0.03	0.02	0.02	0.02	0.02	0.02	0.03	0.03
$\sigma_{a}^{2}$	9.5	9.7	684.6	704.2	47.9	49.1	0.98	1.08	8.85	8.99	789.78	785.59	49.81	49.87	1.31	1.31
$\sigma_{r}^{2}$	14.2	14.1	1407.5	1391.6	83.7	82.7	1.84	1.76	14.18	14.08	1220.2	1222.4	78.7	78.64	1.52	1.52

ST: Single-trait; MT: Multi-trait; BFT: Back fat thickness; CWT: Carcass weight; EMA: Eye muscle area; MS: Marbling score; $\sigma_{a}^{2}$: Additive genetic variance; $\sigma_{r}^{2}$: Residual variance, $h^{2}$: Heritability, s.e: Standard error.

## References

[1] Lee S.-H., Park B.-H., Sharma A., Dang C.-G., Lee S.-S., Choi T.-J., Choy Y.-H., Kim H.-C., Jeon K.-J., Kim S.-D. (2014). Hanwoo cattle: Origin, domestication, breeding strategies and genomic selection. J. Anim. Sci. Technol..

[2] Soller M., Beckmann J. (1983). Genetic polymorphism in varietal identification and genetic improvement. Theor. Appl. Genet..

[3] Calus M.P., Veerkamp R.F. (2011). Accuracy of multi-trait genomic selection using different methods. Genet. Sel. Evol..

[4] Jia Y., Jannink J.-L. (2012). Multiple-Trait Genomic Selection Methods Increase Genetic Value Prediction Accuracy. Genetics.

[5] Guo G., Zhao F., Wang Y., Zhang Y., Du L., Su G. (2014). Comparison of single-trait and multiple-trait genomic prediction models. BMC Genet..

[6] Christensen O.F., Madsen P., Nielsen B., Ostersen T., Su G. (2012). Single-step methods for genomic evaluation in pigs. Animal.

[7] Åkesson M., Bensch S., Hasselquist D., Tarka M., Hansson B. (2008). Estimating heritabilities and genetic correlations: Comparing the ‘animal model’with parent-offspring regression using data from a natural population. PLoS ONE.

[8] Hayes B., Goddard M. (2001). Prediction of total genetic value using genome-wide dense marker maps. Genetics.

[9] Ayalew W., Aliy M., Negussie E. (2017). Estimation of genetic parameters of the productive and reproductive traits in Ethiopian Holstein using multi-trait models. Asian Australas. J. Anim. Sci..

[10] Sawyer S.F. (2009). Analysis of variance: The fundamental concepts. J. Man. Manip. Ther..

[11] Agresti A. (1990). Categorical Data Analysis.

[12] Misztal I., Tsuruta S., Lourenco D., Aguilar I., Legarra A., Vitezica Z. (2014). Manual for BLUPF90 Family of Programs.

[13] Misztal I., Tsuruta S., Strabel T., Auvray B., Druet T., Lee D. BLUPF90 and related programs (BGF90). Proceedings of the 7th World Congress on Genetics Applied to Livestock Production.

[14] VanRaden P.M. (2008). Efficient methods to compute genomic predictions. J. Dairy Sci..

[15] Guo X., Christensen O.F., Ostersen T., Wang Y., Lund M.S., Su G. (2015). Improving genetic evaluation of litter size and piglet mortality for both genotyped and nongenotyped individuals using a single-step method. J. Anim. Sci..

[16] Choi T., Alam M., Cho C., Lee J., Park B., Kim S., Koo Y., Roh S. (2015). Genetic parameters for yearling weight, carcass traits, and primal-cut yields of Hanwoo cattle. J. Anim. Sci..

[17] Lee S.H., Choi B.H., Lim D., Gondro C., Cho Y.M., Dang C.G., Sharma A., Jang G.W., Lee K.T., Yoon D. (2013). Genome-wide association study identifies major loci for carcass weight on BTA14 in Hanwoo (Korean cattle). PLoS ONE.

[18] Park B., Choi T., Kim S., Oh S.-H. (2013). National genetic evaluation (system) of Hanwoo (Korean native cattle). Asian-Australas. J. Anim. Sci..

[19] Bhuiyan M., Kim H., Lee D., Lee S., Cho S., Yang B., Kim S., Lee S. (2017). Genetic parameters of carcass and meat quality traits in different muscles (longissimus dorsi and semimembranosus) of Hanwoo (Korean cattle). J. Anim. Sci..

[20] Oikawa T., Sanehira T., Sato K., Mizoguchi Y., Yamamoto H., Baba M. (2000). Genetic parameters for growth and carcass traits of Japanese Black (Wagyu) cattle. Anim. Sci..

[21] Júnior G.A.F., Rosa G.J., Valente B.D., Carvalheiro R., Baldi F., Garcia D.A., Gordo D.G., Espigolan R., Takada L., Tonussi R.L. (2016). Genomic prediction of breeding values for carcass traits in Nellore cattle. Genet. Sel. Evol..

[22] Do C., Park B., Kim S., Choi T., Yang B., Park S., Song H. (2016). Genetic parameter estimates of carcass traits under national scale breeding scheme for beef cattle. Asian-Australas. J. Anim. Sci..

[23] Conner J.K. (2012). quantitative genetic approaches to evolutionary constraint: how useful?. Evolution.

[24] Hayashi T., Iwata H. (2013). A Bayesian method and its variational approximation for prediction of genomic breeding values in multiple traits. BMC Bioinform..

